# The associations between sleep quality, mood, pain and appetite in community dwelling older adults: a daily experience study

**DOI:** 10.1016/j.jnha.2023.100028

**Published:** 2024-01-01

**Authors:** Hanneke A.H. Wijnhoven, Almar A.L. Kok, Laura A. Schaap, Trynke Hoekstra, Maartje M. van Stralen, Jos W.R. Twisk, Marjolein Visser

**Affiliations:** a Department of Health Sciences, Faculty of Science, Amsterdam Public Health Research Institute, Vrije Universiteit Amsterdam, Amsterdam, The Netherlands; b Department of Epidemiology and Data Science, Amsterdam Public Health Research Institute, Methodology Programme, Amsterdam UMC, Location VU University Medical Center, Amsterdam, The Netherlands; c Department of Psychiatry, Amsterdam Public Health Research Institute, Aging & Later Life Programme, Amsterdam UMC, Location VU University Medical Center, Amsterdam, The Netherlands

**Keywords:** Experience sampling, Anorexia, Malnutrition, Depression, Determinants

## Abstract

**Objectives:**

To investigate the daily life experiences of sleep, mood, and pain in relation to appetite in community-dwelling older adults aged 75 years and older, stratified by sex.

**Design:**

Existing data from a daily experience study embedded in the Longitudinal Aging Study Amsterdam (LASA) among the oldest-old (≥75 years).

**Setting:**

LASA is an ongoing cohort study of a nationally representative sample of older adults aged ≥55 years from three culturally distinct regions in the Netherlands.

**Participants:**

434 community-dwelling older adults aged ≥75 years.

**Measurements:**

Participants filled-out a one-week diary on daily experience of pain, mood, last night sleep (10-point Likert scale), and appetite (5-point Likert scale) on five measurement occasions between 2016 and 2021. (Hybrid) linear mixed models were used to investigate overall, within-subject and between-subject association between mood, sleep, and pain (independent variables) and appetite (dependent variable), while correcting between-subject associations for season, age, educational level, partner status, body mass index, alcohol consumption, physical activity level, smoking status, chronic diseases and use of nervous system medication, stratified by sex.

**Results:**

Averaged over all days, males reported a poor appetite on 12% of the days and females on 19% of the days. Statistically significant between-subject associations with a poorer appetite were found for lower mood (unstandardized b = 0.084 [95% CI 0.043–0.126] (males), (b = 0.126 [95% CI 0.082–0.170] (females)), poorer sleep (b = 0.045 [95% CI 0.007–0.083] (males), (b = 0.51 [95% CI 0.017–0.085] (females)) and more severe pain in males only (b = 0.026 [95% CI 0.002–0.051]). Except for pain, within-subject associations were somewhat weaker: mood: b = 0.038 [95% CI 0.016–0.060] (males), (b = 0.082 [95% CI 0.061–0.104] (females)); sleep: b = 0.029 [95% CI 0.008–0.050] (males), (b = 0.15 [95% CI 0.005–0.025] (females)); and pain (b = 0.032 [95% CI 0.004–0.059] (males)).

**Conclusions:**

This study found that poor sleep, low mood (more strongly in females) and more severe pain (males only) are associated with poor appetite in older adults on a daily level both within and between persons. Sex differences in factors related to poor appetite should be considered in future research.

## 1 Background and objectives

Older adults frequently face a decline in appetite [1] which increases the risk of malnutrition [[2], [3], [4]], frailty [5] and mortality [6]. In community-dwelling older populations, the prevalance of poor appetite varies between 11 and 27% [[7], [8], [9], [10], [11], [12]]. The causes of this age-related decrease in appetite remain unclear but are likely multi-factorial, involving physiological changes associated with aging, pathological conditions, and social factors [13]. To develop interventions that promote appetite in old age, a better understanding of potentially modifiable factors contributing to poor appetite is necessary.

Numerous prior cross-sectional studies have identified a broad range of social, health related, functional, emotional, cognitive and lifestyle factors associated with poor appetite in older adults. These include older age [[14], [15], [16]], female sex [10,12,15,16], living alone [14], lower socio-economic status or education [11,15,16], chewing problems [10,12,14,17], poor oral health [16,17], olfactory dysfunction [18], visual impairment [12], (unintended) weight loss [10,12], presence of sarcopenia [15], higher plasma concentration of tumour necrosis factor-alpha [12], polypharmacy [10,15], presence of pressure ulcers [14], presence of a chronic disease [14], poor self-reported health [12], chronic pain [19], impairment in activities of daily living [11,14,16], depressive symptoms [10,12,14,20,21], hardiness (ability to resist illness when under stress) [20], lower mood [20], lower cognitive function [[14], [15], [16]], current smoking [12,15], physical inactivity [15], and sleep problems [22,23]. Some of these studies employed a univariable approach [[14], [15], [16], [17]^19^] while others adjusted for other risk factors [10,12,18,[20], [21], [22], [23]].

However, these earlier studies share a common limitation in that they rely on cross-sectional analyses of single-point (retrospective) assessments. While this approach is suitable for examining between-subject associations, it fails to capture potential day-to-day fluctuations within individuals. Moreover, single-point retrospective assessments can be influenced by recall bias [24]. To overcome these limitations, experience sampling methodology, also known as ecological momentary assessment or daily diary methodology, has gained popularity. This approach involves collecting repeated, usually daily, measurements, minimizing recall bias and accounting for day-to-day fluctuations [25]. One other key advantage is that it captures individuals in their natural environments, enhancing the relevance of findings to real-world situations [26]. Furthermore, this longitudinal design allows the separation of between-subject and within-person associations. Examining within-person associations methodologically ensures that effect estimates remain unaffected by (stable) differences between subjects.

To date, no study has employed experience sampling methodology to investigate factors related to appetite in older adults. Previous research has linked poor appetite to depressive symptoms [10,12,14,20,21] or lower mood [20], and few studies found associations with sleep problems [22,23] and pain [19]. These factors often exhibit day-to-day variations and are known to correlate at the daily level [27,28]. It is, however, unclear whether appetite varies from day to day and whether this variation is associated with daily experiences of sleep, mood, and pain. Therefore, this study aims to investigate these associations among community-dwelling older adults using existing data from a daily experience study integrated into the Longitudinal Aging Study Amsterdam (LASA). Additionally, we will explore potential sex differences in appetite associations, recognizing the importance of considering sex and gender differences in health research [29], with only two prior studies having examined sex differences in factors associated with appetite [15,22].

## 2 Methods

### 2.1 Design and participants

The design of the study is a (longitudinal) daily experience study making use of data collected by daily diaries. This daily experience study was embedded in LASA. LASA is an ongoing cohort study of a nationally representative sample of older adults aged ≥55 years from three culturally distinct regions in the Netherlands, focusing on the determinants, trajectories and consequences of physical, cognitive, emotional and social functioning [30,31]. Participants are visited every 3–4 years at home by trained interviewers and data are collected by means of face-to-face interviews, clinical measurements and self-administered questionnaires. Ethical approval for the LASA study was given by the Institutional Review Board of the VU University Medical Centre Amsterdam (METC number 2012/361), and all participants provided written informed consent.

We analysed data from a daily experience study that was embedded within the ancillary 75PLUS study conducted among the oldest-old individuals (aged ≥75 years) of LASA. As part of this study, participants completed a one-week diary, documenting their daily experiences of various factors on five different measurement occasions (t1-t5) between 2016 and 2021. The first three one-week diaries (t1-t3) were filled out during three additional nine-monthly measurements conducted between the regular measurement waves of 2015/16 and 2018/19, the fourth diary (t4) was filled out during the regular 2018/19 wave and the fifth diary (t5) 23 months after the regular 2018/19 wave. A detailed description of this ancillary study can be found elsewhere [30]. In brief, all LASA participants aged 75 years and over (born before 1941) at the 2015/16 wave were invited to participate in the 75PLUS study (n = 686). Respondents who underwent an additional face-to-face interview as part of this study were asked to fill out a one-week diary to study changes in pain, use of pain medication, mood, sleep, appetite, and number of social contacts on a daily basis and return the diary by postal mail. Of the eligible participants, 387 returned the diary at t1, 368 at t2, 325 at t3 and 311 returned the diary at t4. For t5, all LASA participants aged 75 years and over (born before 1946) at the regular 2018/19 wave and participating in the regular face-to-face interview were invited (n = 525) of whom 434 persons agreed to participate. Those who had previously filled out a one-week diary were asked to fill out a week diary which resulted in 214 diaries returned at t5. In total, 445 participants returned at least one of the five week diaries. For the present analyses, we only included week diaries with complete data for mood, sleep, pain and appetite for at least three days in one week. The final analytical sample for the present study thereby included 434 participants who completed in total 3.5 (SD 1.4) week diaries and 10453 measurement days. The selection of the study sample is described in Supplementary Table 1.

### 2.2 Week diaries

Participants received a one-week diary on paper, on which they were asked to record for 7 consecutive days their experience of e.g. pain, mood, sleep, and appetite on a Likert scale. For pain the question posed was: “How much pain did you experience today? (1 = severe pain, 10 = no pain at all). Answers were recorded as an integer. For the the fifth diary (t5), the pain (answer) scale was reversed for participants and reversed back for the analyses. For mood, the question posed was: “How was your mood today? (1 = a very poor mood, 10 = a very good mood). For sleep, the question posed was: “How well did you sleep last night? (1 = very poorly, 10 = very well). For appetite, the question posed was: “How do you rate your appetite today? (Very poor/poor/moderate/good/very good). This was coded on a scale from 1 to 5 for the analyses.

### 2.3 Other measurements

Descriptive characteristics and potential confounding variables of between-subject associations were (except for season and age) based on data collected during the regular measurement 2015/16 and 2018/19. Sex was defined as male or female. Age was calculated by the number of days between the date of the main interview in 2015/16 (for descriptives) or the date of the week diary measurement and the birth date and expressed in years. Season was based on the date of the week diary measurement and defined as spring/summer versus autumn/winter. Education was categorized into low (elementary education or less), middle (lower vocational education and general intermediate education) and high education (intermediate vocational education, general secondary education, higher vocational education, college education and university). Living situation was defined as living alone or living with partner/spouse/others. Alcohol consumption was expressed by the average number of alcohol drinks per day and categorized into none, up to two and more than two. Smoking status was defined as never, former and current smoker. Physical activity was assessed by the LASA Physical Activity Questionnaire and expressed as total metabolic equivalent of task (MET) hours/week, including walking outdoors, light household activities, heavy household activities and two most frequently performed sports performed in the past two weeks [32,33]. Body mass index (BMI) was calculated by dividing body weight in kilograms by body height in meters squared. Body weight was measured to the nearest 0.1 kg using a calibrated bathroom scale (Seca, model 100, Lameris, Utrecht, The Netherlands). Body height was measured to the nearest 0.1 cm using a stadiometer. If missing, self-reported body weight (approximately 1%) and body height (approximately 2%) were used. Self-reported chronic disease was defined as the number of chronic diseases of the most frequently occurring somatic chronic disease in the Netherlands: chronic non-specific lung disease, cardiac disease, peripheral artery diseases, diabetes mellitus, cerebrovascular accident or stroke, osteoarthritis, rheumatoid arthritis and/or cancer and a maximum of two other chronic diseases which symptoms lasted for at least three months. The total number of chronic diseases was recoded into a score that ranged from 0 (no chronic disease) to 3 (three or more chronic diseases). Use of nervous system medication in the past week (yes or no) was defined as the use of physician prescribed medicines that fall under the Anatomical Therapeutic Chemical code nervous system (e.g., antidepressants, anxiolytics, hypnotics).

### 2.4 Statistical analyses

Descriptive baseline information of the analytical sample and the total eligible LASA sample aged ≥75 years was presented stratified by sex as means with standard deviations (±SD) or as frequencies, using data of the regular wave 2015/16. Pooled summary statistics (means with SD and interquartile range and - for appetite only - frequencies) were described for each daily measure (mood, sleep, pain, and appetite), stratified by sex. In addition, the within-person variation of all daily measures during a week was calculated by subtracting the participants’ score on each day from the participants’ mean score over 7 days, expressed in absolute values. The pooled mean of this deviation score was also described, stratified by sex.

Linear mixed model analyses for repeated measures with appetite score as dependent variable were used to investigate the longitudinal associations of mood, sleep, and pain with appetite. Linear mixed models can be used to analyse Likert scale data [34,35]. Because few participants had a very poor appetite, the very poor and poor categories were merged. Besides the overall longitudinal association, hybrid mixed models as described by Twisk and de Vente were used to separately estimate between-person and within-person associations [36]. To accomplish this, we computed two new variables for each independent variable mood, sleep and pain score. To estimate the between-person associations, we calculated the mean score over 7 days (or less in case of missing days) for each independent variable and each participant and each diary. To estimate the within-person associations, we calculated a deviation score for each daily measurement by subtracting the participants’ score on each day from the participants’ mean score over 7 days. Both the time-independent mean score and the time-dependent deviation score were simultaneously inserted as independent variables in the hybrid mixed model. The regression coefficient of the mean score reflects the between-subject associations, indicating if differences between participants' appetite score are related to differences between participants' mood, sleep and pain scores. The regression coefficient of the deviation score reflects the within-subject associations, indicating if differences within participants’ appetite score are related to differences within participant’s mood, sleep and pain scores. Within the mixed models, we applied a three-level hierarchical structure with daily measurements clustered within measurement occasion (5 weekly diaries) and measurement occasions clustered within participants.

For each independent variable (mood, sleep, pain), we applied unadjusted overall (non-hybrid) models and hybrid models. We subsequently adjusted all models for: (1) the other independent variables; (2) potential confounding variables: season (spring/summer versus autumn/winter), demographics (age, education, partner status), lifestyle factors (BMI, alcohol use, physical activity, smoking), and health-related factors (number of chronic diseases and use of nervous system medication). For the first three-week diaries, potential confounders measured at the 2015/16 wave were used and for the last two week diaries confounders measured at the 2018/19 wave were used. All models were presented stratified by sex and tested for interaction by sex. All models included a random intercept on both measurement occasion and participant level and a random slope for the main independent variables mood, sleep and pain (either overall, within-person, or between-person) on participant level. For the covariates only fixed effects (i.e., no random slopes) were modelled. Linear mixed model analyses were performed using STATA (version 17.0).

## 3 Results

At baseline, the analytical sample (n = 434) included more females (59%) than males with an approximately similar mean age (81 ± 5 years) (Table 1). Compared to males in the study sample, females were lower educated, more often lived alone, had a comparable BMI, drank less alcohol, smoke(d) less often, were more often physically active, and had more chronic diseases. Excluded (eligible) males and females were older, lower educated and more often lived alone. Furthermore, although interpretation may be hampered by missing observations, they had a lower BMI (for males only), drank less alcohol, were more often former smoker (males) or never smoker (females), were less physically active, and had (slightly) more chronic diseases (in males only).

**Table 1:** Baseline characteristics (2015/16) of the included and excluded sample of the oldest-old (≥75 years) participants of the Longitudinal Aging Study Amsterdam, stratified by sex.

	Included analytical sample (n = 434)		Excluded eligible sample (n = 252)	
	Males	Females	Males	Females
N	179	255	98	154
Age (years)	80.9 ± 4.9	81.5 ± 4.9	84.7 ± 5.4	85.6 ± 6.2
Education				
Low	12.3	23.1	25.5	48.1
Middle	27.9	43.1	32.7	35.1
High	59.8	33.7	41.8	16.9
Living situation (living with partner)	72.6	34.1	55.1	20.8
Body mass index (kg/m^2^)	(n = 171) 27.0 ± 4.2	(n = 242) 27.2 ± 6.1	(n = 39) 24.2 ± 7.9	(n = 55) 27.1 ± 5.8
Alcohol consumption	(n = 170)	(n = 242)	(n = 39)	(n = 55)
None	14.7	25.2	23.1	40.0
Light/moderate (up to 2 per day)	53.5	63.6	66.7	52.7
Heavy/extreme (more than 2 per day)	31.8	11.2	10.3	7.3
Smoking status	(n = 171)	(n = 242)	(n = 39)	(n = 55)
Never	13.5	40.9	5.1	56.4
Former	77.8	52.2	87.2	38.2
Current	13.5	5.9	7.7	5.5
Physical activity (MET hours per week)^a^	(n = 176) 44.3 ± 30.5	(n = 251) 51.5 ± 32.7	(n = 53) 34.5 ± 30.9	(n = 75) 38.5 ± 25.0
Chronic diseases (self-reported)	(n = 176)	(n = 252)	(n = 55)	(n = 76)
None	9.1	6.0	5.5	6.6
One	19.9	16.7	16.4	13.2
Two	25.6	26.6	27.3	26.3
Three or more	45.5	50.8	50.9	53.9

Data are presented as mean ± standard deviation, or percentage (%).

a Including walking outdoors, light and heavy household activities and two most frequency performed sports.

In the total analytical dataset, there were 4450 daily observations for males and 6003 observations for females (Table 2). Averaged over all days, males reported a poor appetite on 12% of the days and females on 19% of the days. The average scores for mood, last night sleep and pain were relatively high (around 8) indicating a relatively good mood, good last night sleep and absence of severe pain. All scores were on average somewhat lower for females. The SD was highest for pain indicating that pain scores varied somewhat more between and/or within respondents compared to mood and last night sleep. The absolute mean deviation scores were, except for appetite, somewhat higher for females than for males, suggesting a higher within-person variation over the week for females.

**Table 2:** Summary statistics for the daily measures on experience of mood, last night sleep, pain, and appetite across all daily observations, stratified by sex.

	Males (n observations = 4450)					Females (n observations = 6003)				
Daily measure	%	Mean	SD	IQR	Mean deviation^c^	%	Mean	SD	IQR	Mean deviation^c^
Mood^a^		8.1	1.3	7−9	0.35		7.7	1.2	7−8	0.41
Last night sleep^a^		7.9	1.6	7−9	0.44		7.2	1.7	6−8	0.66
Pain^a^		8.5	1.9	8−10	0.30		7.6	2.2	6−10	0.48
Appetite^b^		4.0	0.6	4−4	0.11		3.9	0.6	4−4	0.12
Appetite score:										
Very poor	0.0					0.2				
Poor	1.6					3.2				
Moderate	9.9					15.6				
Good	74.8					72.6				
Very good	13.7					8.4				

SD = Standard deviation IQR = Interquartile range (25^th^–75^th^ percentile).

a Assessed on a 10-point Likert scale from 1 (severe pain/very low mood/very poor sleep) to 10 (no pain/very good mood/very good sleep).

b Assessed on a 5-point Likert scale (1 = very poor/2 = poor/3 = moderate/4 = good/5 = very good).

c Absolute deviation score, calculated by subtracting the participants’ score on each day from the participants’ mean score over 7 days.

Results of the univariable and multivariable (hybrid) mixed models are shown in Table 3. As all independent variables are assessed on a 10-point Likert scale, the effect sizes can be compared directly. Statistically significant between-subject associations with a poorer appetite were found for lower mood (unstandardized b = 0.084 [95% CI 0.043–0.126] (males), (b = 0.126 [95% CI 0.082–0.170] (females)), poorer sleep (b = 0.045 [95% CI 0.007–0.083] (males), (b = 0.51 [95% CI 0.017–0.085] (females)) and more severe pain in males only (b = 0.026 [95% CI 0.002–0.051]). Within-subject associations were somewhat weaker: mood: b = 0.038 [95% CI 0.016–0.060]. (males), (b = 0.082 [95% CI 0.061–0.104] (females)); sleep: b = 0.029 [95% CI 0.008–0.050] (males), (b = 0.15 [95% CI 0.005–0.025] (females)); and pain (b = 0.032 [95% CI 0.004–0.059] (males)). Interaction with sex was statistically significant (*p* <  0.05) for mood and pain in the overall (non-hybrid) model and for within-subject associations of mood.

**Table 3:** Associations between mood, sleep, and pain (independent variables) with appetite (dependent variable) using (hybrid) mixed models^a^, stratified by sex.

	Males (n observations = 4450)			Females (n observations = 6003)			
	b	95% CI	*p*	b	95% CI	*p*	*p* interaction^b^
**Univariable models**							
Mood_overall_	0.074	0.052–0.096	**<.001**	0.100	0.081–0.120	**<.001**	0.077
Mood_within_	0.063	0.039–0.087	**<.001**	0.090	0.069–0.111	**<.001**	0.150
Mood_between_	0.127	0.093–0.162	**<.001**	0.162	0.125–0.198	**<.001**	0.224
Sleep_overall_	0.052	0.036–0.070	**<.001**	0.037	0.026–0.048	**<.001**	0.362
Sleep_within_	0.043	0.020–0.066	**<.001**	0.030	0.018–0.041	**<.001**	0.299
Sleep_between_	0.097	0.065–0.129	**<.001**	0.106	0.074–0.139	**<.001**	0.555
Pain_overall_	0.043	0.022–0.065	**<.001**	0.021	0.009–0.032	**<.001**	0.096
Pain_within_	0.031	0.003–0.059	**0.033**	0.018	0.002–0.033	**0.019**	**0.032**
Pain_between_	0.059	0.037–0.080	**<.001**	0.029	0.013–0.046	**<.001**	0.169
**Multivariable models**^c^							
Mood_overall_	0.052	0.032–0.072	**<.001**	0.092	0.073–0.111	**<.001**	**0.025**
Mood_within_	0.041	0.019–0.063	**<.001**	0.083	0.071–0.095	**<.001**	**0.039**
Mood_between_	0.088	0.047–0.129	**<.001**	0.126	0.092–0.160	**<.001**	0.334
Sleep_overall_	0.033	0.015–0.051	**<.001**	0.052	0.023–0.080	**<.001**	0.195
Sleep_within_	0.031	0.010–0.052	**0.004**	0.015	0.007–0.022	**<.001**	0.216
Sleep_between_	0.047	0.011–0.083	**0.011**	0.051	0.017–0.085	**0.003**	0.757
Pain_overall_	0.029	0.009−0.049	**0.005**	0.001	−0.009−0.010	0.911	**0.047**
Pain_within_	0.025	−0.003–0.052	0.076	−0.001	−0.011−0.009	0.837	0.218
Pain_between_	0.027	0.005-0.049	**0.018**	0.005	−0.012−0.009	0.591	0.212
**Multivariable models**^d^							
Mood_overall_	0.048	0.028–0.069	**<.001**	0.091	0.071–0.111	**<.001**	**0.018**
Mood_within_	0.038	0.016–0.060	**0.001**	0.082	0.061–0.104	**<.001**	**0.030**
Mood_between_	0.084	0.043–0.126	**<.001**	0.126	0.082–0.170	**<.001**	0.330
Sleep_overall_	0.045	0.072–0.083	**0.019**	0.019	0.010–0.028	**<.001**	0.186
Sleep_within_	0.029	0.008–0.050	**0.007**	0.015	0.005–0.025	**0.003**	0.188
Sleep_between_	0.045	0.007–0.083	**0.004**	0.051	0.017-0.085	**0.003**	0.788
Pain_overall_	0.032	0.010–0.053	**0.004**	0.001	−0.010−0.011	0.904	**0.039**
Pain_within_	0.032	0.004–0.059	**0.023**	0.000	−0013−0.013	0.972	0.140
Pain_between_	0.026	0.002–0.051	**0.035**	−0.008	−0.025−0.009	0.364	0.210

B = unstandardized regression coefficient. CI = Confidence interval.

Bold = statistically significant (*p* < 0.05).

a All models included a random intercept on both measurement occasion and participant level and a random slope for the main independent variables mood, sleep and pain on participant level. For the covariates only fixed effects (i.e., no random slopes) were modelled.

b Testing for interaction by sex.

c Mood, sleep and pain included simultaneously.

d Mood, sleep and pain included simultaneously and adjusting for season, age, education, partner status, body mass index, alcohol, physical activity, smoking, chronic diseases and use of nervous system medication.

## 4 Discussion

This study is the first to explore how daily life experiences of sleep, mood and pain relate to appetite using experience sampling methodology in a cohort of community dwelling older adults aged 75 years and older. Males reported a poor appetite on 12% of the measured days and females on 19% of the days. We found that those with lower mood or poorer sleep, and males with more severe pain, have a poorer appetite compared to those with higher mood, better sleep, or less pain. Additionally, a decrease in mood, deterioration of last night's sleep, or an increase in pain (males only) are associated with a decrease in appetite on the same day. Sex differences were observed, with mood being more strongly associated with appetite in females and the association with pain being only observed in males.

Of all factors, depressive symptoms have been most often studied and linked to a poorer appetite in older adults in cross-sectional studies [10,12,14,20,21]. In our study, among the three factors examined, mood showed the strongest association with a poor appetite. It is important to clarify that a single question about experienced mood is not equivalent to a full assessment of depressive symptoms. However, a cross-sectional study among 292 older adults (≥60 years) attending senior centres or living in assisted living facilities found that a single-item question about mood was even more strongly correlated with a poor appetite than using the 5-item Geriatric Depression Scale to assess depressive symptoms [20]. The association between mood and appetite may be explained by the effect of mood on taste and smell reduction [37], induction of anhedonia [38], or reduction of executive functioning associated with fatigue [39], subsequently lowering appetite. Though reduced taste and smell may remain stable from day-to-day, the latter two pathways might explain within-subject associations. We also found that the mood-appetite association is stronger in older females than in older males. Prior studies did not stratify by sex, so comparisons are limited. A recent qualitative study among older adults revealed that mood significantly influences the emotional experience of appetite and that older females often discussed various mood states related to poor appetite, while older males related their poor appetite experiences to clinical depression or stress [40]. If confirmed by other studies, our findings suggest that older females may be more vulnerable to mood's impact on appetite than older males.

Our study aligns with previous cross-sectional research, which suggests a link between sleep problems and poor appetite in older adults, even when adjusting for depressive symptoms [22,23]. However, one study found a positive association that lost statistical significance after accounting for various confounding factors [10], likely due to lower statistical power compared to our study. Furthermore, recent studies have linked poor sleep quality or shorter sleep duration with malnutrition [[41], [42], [43], [44], [45], [46]], possibly mediated by poor appetite. Several hypotheses can be proposed to explain the underlying mechanism. One is that between-subject associations may be attributed to lower expression of the neuropeptide orexin with aging in some individuals, leading to both reduced appetite and lower daytime arousal [47,48]. Consequently, increased daytime napping could negatively impact nocturnal sleep [49]. However, this explanation is less likely to explain the day-to-day within-subject associations. Another possibility is that poor appetite leads to inadequate nutritional intake, which in turn results in poorer sleep due to reduced levels of sleep-inducing hormones like serotonin and melatonin, both derived from tryptophan. However, the most plausible explanation appears to be that poor sleep causes fatigue the following day, subsequently reducing appetite. Regarding the presence of sex differences, our study did not uncover strong evidence supporting their existence.

We identified only two prior cross-sectional studies that examined the association between (chronic) pain and appetite in older adults [10,19]. In one of these studies, an initial univariable analysis revealed an association between the presence of pain and poor appetite. However, this association lost statistical significance after adjusting for a broad range of confounding factors [10]. In the other study, which focused on 65 older adults with non-malignant pain (75% females), it was found that those reporting poorer appetite also reported higher pain intensity [19]. However, no statistical adjustments were made for other factors. The link between pain and reduced appetite might be explained by a neural mechanism. Studies conducted on rats suggest that specific hypothalamic neurons, responsible for suppressing appetite in response to metabolic cues, may also play a role in reducing food intake in response to pain signals [50]. Notably, while reports of pain are consistently higher in females, we observed associations between pain and appetite in older males, both between subjects and within subjects, but not in older females. This could be due to to sex differences in, e.g., pain perception or psychological and emotional responses to pain that impact appetite regulation. However, further research is required to validate these findings.

This study is the first to explore appetite associations by using experience sampling methodology and systematically considering potential sex differences. Our study design not only reaffirmed the associations between factors previously linked to poor appetite in cross-sectional studies at the between-subject level, accounting for confounding factors, but also identified daily within-individual associations. Furthermore, in addition to the more established association between mood and appetite, our study also shed light on the less-explored association between sleep and pain with appetite.

The study's strengths include a large sample of older adults from a population-based study and the use of multiple week diaries, which increased study power, enabling detection of small associations. However, limitations should be acknowledged. Participants were somewhat healthier and higher educated, potentially underestimating associations due to lower prevalence of poorer appetite and other experienced problems, reducing measure variance. Additionally, existing data limited the inclusion of all potential time-varying factors. Future studies with similar methodology could consider factors like physical activity and sedentary behaviour that may vary from day-to-day. Moreover, within and between-person associations might be influenced by unknown (confounding) factors, such as hormonal fluctuations or unaccounted external factors like the time of day when participants completed their diaries. Lastly, measurements were conducted only once a day while the experiences of pain, mood, sleep, and appetite may also fluctuate throughout the day.

The results of this study suggest that poor sleep, lower mood (more strongly in females) and more severe pain (males only) are associated with poorer appetite in older adults on a daily level both within and between persons. As a poor appetite is an important risk factor for the development of malnutrition [[2], [3], [4]], frailty [5] and mortality [6], interventions targeting these underlying factors could prevent or postpone these adverse outcomes. In addition, clinicians should be aware that a poor appetite can signal the presence of other underlying issues such as low mood, poor sleep, or pain, all of which have a substantial impact on an individual's quality of life and necessitate attention. Additionally, the observed sex differences suggest that factors linked to poor appetite may vary based on sex. This highlights the importance of considering sex differences in both future research and, if confirmed by other studies, clinical practice. Such an approach can lead to more tailored and effective interventions to address appetite-related concerns among older adults.

## Funding

The Longitudinal Aging Study Amsterdam is supported by a grant from the Netherlands Ministry of Health Welfare and Sports, Directorate of Long-Term Care.

## Ethical standards

Ethical approval for the Longitudinal Aging Study Amsterdam was given by the Institutional Review Board of the VU University Medical Centre Amsterdam (METC number 2012/361), and all participants provided written informed consent.

## Conflict of interest

All authors (Hanneke A.H. Wijnhoven, Almar A.L. Kok, Laura A. Schaap, Trynke Hoekstra, Maartje M. van Stralen, Jos W.R. Twisk, Marjolein Visser) have no conflicts of interest to declare.
