# Supplementary material for: The associations between sleep quality, mood, pain and appetite in community dwelling older adults: a daily experience study
Source: J Nutr Health Aging. 2024 Jan 1;28(2):100028. doi: 10.1016/j.jnha.2023.100028 (PMC12877243; doi:10.1016/j.jnha.2023.100028)
Supplement: Supplementary file 1 [file mmc1.docx]

**Supplementary Table 1.** Daily experience study among the oldest old (aged ≥ 75 years) participants of the Longitudinal Aging Study Amsterdam (LASA): selection of study sample.

| 75PLUS study: | t1 | t2 | t3 | t4 | t5 |
| --- | --- | --- | --- | --- | --- |
| Date range interviews: | Jul. 2016-  Jul. 2017 | Apr. 2017-  Apr. 2018 | Jan. 2018-  Jan. 2019 | Oct. 2018-  Oct. 2019 | Sep 2020-  Feb 2021 |
| Invited for ancillary study | 686 | 601 | 550 | 473 | 525 |
| Invited for diary study | 442 | 410 | 364 | 359^1^ | 238 |
| Returned week diary | 387 | 368 | 325 | 311 | 214 |
| Included in analytical sample^2^ | 372 | 338 | 318 | 299 | 198 |

^1^ Only respondents who previously filled-out a week-diary were invited to fill out a week diary

^2^ Respondents with complete data for mood, sleep, pain and appetite for at least three days in one week.
